# *Elizabethkingia meningosepticum* (*Chryseobacterium meningosepticum*) Infections in Children

**DOI:** 10.1155/2011/215237

**Published:** 2011-10-20

**Authors:** Mehmet Ceyhan, Melda Celik

**Affiliations:** Department of Pediatric Infectious Diseases, Hacettepe University Faculty of Medicine, 06100 Ankara, Turkey

## Abstract

*Chryseobacterium meningosepticum* is a ubiquitous Gram-negative bacillus historically associated primarily with meningitis in neonates and a wide variety of infections in immunocompromised patients. Neonatal infections often occur as outbreaks with environmental contamination being the source. *C. meningosepticum* infections are not common but are clinically important because the organism is naturally resistant to multiple antibiotics. In this paper, we have reviewed the nosocomial outbreaks of *C. meningosepticum* in newborns and infants reported so far in the literature and overviewed the infection control interventions, treatment modalities, and prevention measures.

## 1. Introduction

*Chryseobacterium meningosepticum* is a nonfermenting, nonmotile, oxidase-positive Gram-negative aerobic bacillus that is ubiquitous in the environment, found in freshwater, saltwater, and soil. It was first defined by King in 1959 [1]. King also used serological procedures for typing strains isolated in epidemiological studies, and 6 serotypes (A to F) have been described, type C being responsible for most of the cases of meningitis [1]. This ubiquitous bacterium, formerly known as *Flavobacterium meningosepticum* and recently termed *Elizabethkingia meningosepticum (or meningoseptica*) by some authors, belongs to the family of Flavobacteriae and inhabits natural and hospital environments [2–5]. 

 Environmental studies have revealed that the organism can survive in chlorine-treated municipal water supplies, often colonises sink basins and taps, and has become a potential reservoir for infections in the hospital environment [6, 7]. Colonization of patients via contaminated medical devices involving fluids (respirators, intubation tubes, mist tents, humidifiers, incubators for newborns, ice chests, syringes, etc.) has been documented [8, 9]. Contaminated surgically implanted devices such as intravascular catheters and prosthetic valves have also been reported as reservoirs [10].

## 2. Clinical Presentation

In clinical settings, *Chryseobacteria* have been described as etiological agents of meningitis, sepsis, bacteremia, pneumonia, endocarditis, infections of skin and soft tissue, wound infection, abdominal abscess, ocular infections, sinusitis, bronchitis, epididymitis, dialysis-associated peritonitis, and prosthesis-associated septic arthritis [6, 11–13]. As primarily opportunistic pathogens, they infect mainly newborns and immunocompromised hosts from all age groups [6].

 Among the *Chryseobacterium* species, *C. meningosepticum* is the most pathogenic member of genus. *C. meningosepticum* is a cause of neonatal meningitis, especially in premature infants during the first weeks of life. In newborns, meningitis is the most common disease caused by this organism. Bacteremia and pneumonia are the other common manifestations in neonates. Infections usually affect premature infants and often occur as outbreaks [9, 14]. Prematurity is a primary risk factor for *C. meningosepticum* infection, and half of the infections have involved neonates weighing less than 2,500 g [9]. Among the various serotypes of *C. meningosepticum* (A to F), type C has been the cause of most reported epidemics [3, 15]. As an agent of neonatal meningitis, it reportedly demonstrates mortality rates up to 57% and produces severe postinfectious sequelae including brain abscesses, hydrocephalus, deafness, and developmental delay. The case-fatality rate has been high in neonates, and early and late complications are common among survivors [11, 15–17]. *C. meningosepticum* is involved to a lesser extent in cases of pneumonia and bacterial sepsis in neonates and adults [6].

 Bacteremia is the second most common presentation of *C. meningosepticum* infection. *C. meningosepticum* has also caused endocarditis (including prosthetic valves), cellulitis, wound infections, sepsis following extensive burns, abdominal abscess, dialysis-associated peritonitis, and endophthalmitis. Infections including cellulitis, septic arthritis, community-acquired respiratory tract infection, keratitis, and bacteremia have been reported in the absence of underlying diseases [18].

## 3. Transmission and Sources of the Infection

 Neonatal infections due to *C. meningosepticum* could be through vertical transmission; however, no data about this kind of transmission of the organism is available. As many interventions (endotracheal intubation, central and peripheral intravascular catheterization, etc.) are made in the neonatal intensive care units (NICUs) and majority of the *C. meningosepticum* infections reported have been in NICUs, the infections of these neonates are often considered as nosocomial. 

 Clusters of neonatal meningitis have been linked to many sources including contaminated saline solution for flushing eyes, respiratory equipment, and sink drains. The respiratory tract is the most common site of infection, and outbreaks have been linked to contaminated ventilator tubing and aerosols. In outbreaks, respiratory tract colonization occurs more often than infection [6]. Other contaminated sources include contaminated syringes in ice chests, vials, sink drains, sink taps, tube feedings, flush solutions for arterial catheters, pressure transducers, and antiseptic solutions [18].

Cabrera and Davis [19] reported an outbreak of 14 cases of neonatal meningitis due to *C. meningosepticum* where the source was traced to a leaking sink trap in an anteroom of the premature ward. Repair of the faulty leaky trap eradicated the reservoir of this infection and terminated the outbreak. Plotkin and McKitrick [20] described two cases of flavobacterium meningitis which were traced to a saline solution. This saline solution containing *Flavobacteria* was stored in a glass bottle that was seldom sterilised but refilled with sterile saline as needed. The solution was aspirated into a rubber bulb and used to flush the eyes of infants after the application of silver nitrate solution. Coyle-Gilchrist et al. [21] isolated *C. meningosepticum* from aqueous chlorhexidine gluconate (Hibitane) solutions used in the wards for the storage of thermometers and for routine disinfection. The two separate *C. meningosepticum* type E outbreaks are reported by Hazuka et al. [15] in the NICU, The organism was first isolated from nasoendotracheal (NET) tube cultures in four of eight infants who were intubated.* C. meningosepticum* was recovered from three nasoendotracheal tubes and from an aerosol tube before colonization of four infants. In the outbreaks, we reported that *C. meningosepticum* was isolated from hand cultures obtained from a senior resident and from environmental cultures obtained from powdered infant formula, an electrical button, a computer keyboard, phone, a doorknob, and an ambu bag.

## 4. Infection and Colonization

 When the organism is colonized in a susceptible individual (e.g., in the respiratory tract), no clinical signs or symptoms are observed, but this person has the potential to infect others unless necessary precautions are taken. Under usual conditions, colonized individuals are not given any antibacterial therapy. If this colonization progresses to clinical infection—demonstrates the signs and symptoms of infection—or the colonized individual infects another susceptible individual and causes infection, then “the infection” should be treated with appropriate antibacterial therapy. 

 Susceptible patients may become colonized after acquiring the organism from the healthcare worker, and infection may or may not ensue [19]. During the outbreak on a NICU, two neonates were infected (one had pneumonia and one septicaemia and meningitis), and six neonates were colonized in the respiratory secretions by a multiresistant *C. meningosepticum. *Two of the eight patients had infection, the others were colonized (in tracheal aspirate, sputum, or perineum) and needed no antibiotic therapy [19]. However, not all neonates exposed to the organism became ill. Cabrera and Galen reported that only one twin of each of three sets became ill and died, while the other twins neither became ill nor were colonised by this organism. This suggests that immune mechanisms may play a role in these nosocomial infections [19]. 

 Maraki et al. [22] reported four neonates with *C. meningosepticum *colonization in the endotracheal tubes and respiratory secretions in an NICU. None of the neonates progressed to clinical infection, and none of them received specific treatment and all survived. This study suggested that *C. meningosepticum* colonization in neonates does not necessarily lead to infection and that such colonization outbreaks may be controlled with emphasis on the standard precautions [22]. If the clinical signs and symptoms can not be evaluated properly, colonization and infection may not be discriminated.

## 5. Hospital Infections

 Since first recognition of *C. meningosepticum* as a cause of neonatal meningitis in 1958, outbreaks have occasionally been described since 1961 [23–28]. 

 Brody et al. [23] described two outbreaks of meningitis caused by *C. meningosepticum* affecting primarily premature infants in a hospital nursery. This outbreak was described as hospital acquired, but attempts to isolate the organism from human contacts were unsuccessful. Cabrera and Galen [19] reported an outbreak of 14 cases of neonatal meningitis, George et al. [29] reported 14 cases of meningitis and 30 asymptomatic nasopharyngeal carriers during an outbreak in their newborn ward. Plotkin and McKitrick [30] described two cases of flavobacterium meningitis which were traced to a saline solution. We could not reach to more detailed data of these reports in our literature search. 

 Between March and July 1975, two separate *C. meningosepticum* type E outbreaks were reported by Hazuka et al. [15] in the neonatal İntensive Care Unit at Children's Hospital of Michigan. Of the 10 infants affected, only 3 in the first outbreak exhibited disease directly related to *C. meningosepticum*. The remaining two infants in March-April and all five infants in July were colonized but not infected. The three ill infants had positive blood cultures. Two developed meningitis; one died within 6 days, and the other survived but developed hydrocephalus requiring a ventriculoperitoneal shunt. The one patient who did not develop meningitis had only a transient bacteremia, was treated with appropriate antibiotics, and eventually was discharged home. The organism was resistant to most antimicrobial agents tested and developed resistance to others during treatment [15].

 Between 1972 and 1977, Thong et al. [14] reported seven infants with *C. meningosepticum* infection. *C. meningosepticum* was isolated from the cerebrospinal fluid in all seven infants, from blood in three infants, and from peritoneal fluid in one infant. Two cases who died did not receive intraventricular chemotherapy. Five infants survived, and three of them were normal neurologically. One of the survivors had hydrocephalus with severe brain damage; the infection in this infant began with umbilical sepsis and peritonitis. The infecting organism was isolated from the peritoneal fluid two days before the cerebrospinal fluid yielded the same organism. The other case also had hydrocephalus without showing any other evidence of neurological handicap [14].

 In 1980, Dooley et al. [30] summarized 63 previously reported cases of *C. meningosepticum* meningitis that occurred from 1944 to 1976. Out of 52 infants for whom the outcome of infection was known, 34 (65%) died. Eleven (61%) of the 18 neonates who survived developed hydrocephalus [31, 32]. Overall, four (50%) of the eight neonates whose length of gestation was known were full-term infants. According to the available data, the mean duration that CSF cultures remained positive was 16 days (range 8–39 days), all of 10 infants had associated bacteremia. One death was related to infection due to *C. meningosepticum*, resulting in a case fatality rate of 8.3%. Hydrocephalus developed in 70% of the surviving infants. Five patients received intrathecal antibiotics [30].

 Di Pentima et al. [33] isolated four strains of *C. meningosepticum* from cerebrospinal fluid and blood from neonates with clinical infection between 1982 and 1996 in Texas Children's Hospital. All of the neonates tolerated treatment with vancomycin and rifampin well, and no adverse effects were noted. All three neonates with meningitis due to *C. meningosepticum *survived without developing hydrocephalus or neurological deficits, as determined on physical examination at the time of discharge. Two infants developed mild sensorineural hearing loss. *C. meningosepticum *was recovered at birth in a tracheal aspirate from the premature infant. Bacteremia due to *C. meningosepticum* subsequently developed in this infant at 3 weeks of age resolved with a 10-day course of vancomycin and ceftazidime treatment. The infant died of *Staphylococcus aureus *septicemia at 2 months of age [33].

 Chiu et al. [7] reported 17 culture-documented systemic infections due to novel, atypical strains of *C. meningosepticum* in two newborns and 15 immunocompromised patients from 1996 to 1999 in a medical center in Taiwan. All clinical isolates were resistant to a number of antimicrobial agents. The isolates were characterized as atypical strains of *C. Meningosepticum,* and that was the first report of a cluster of atypically variant strains of *C. meningosepticum*. Two patients were newborns, and one was 7-year-old child with IgA nephropathy. Three of the 15 nonneonatal patients died of the infection; the two newborns survived with severe neurologic sequelae, despite antibiotic treatment [7].

 Hoque et al. [9] reported a strain of multiresistant *C. meningosepticum *isolated from eight neonates on a neonatal intensive care unit from September 1994 to May 1996. Two neonates were infected (one had pneumonia and one septicaemia and meningitis); the remaining six neonates were colonized in the respiratory secretions. Two cases occurred that could not be explained by cross-infection during the outbreak. The two infected neonates survived, and the neonate with meningitis and septicaemia did not develop hydrocephalus. Both of these infants were very low birth weight neonates (<1500 g) which is consistent with studies that show at least 50% of neonates with this infection weighed <2500 g [9]. 

Güngör et al. [11] reported an outbreak of *C. meningosepticum *in four neonates with sepsis in the NICU of a referral teaching hospital in September 2001. The organism was isolated from blood cultures of all four patients. The first isolate was identified 5 days after the death of the index case. All three patients survived with one having a complication (hydrocephalus) [11]. 

 Maraki et al. [22] reported four neonates with *C. meningosepticum* colonization in the endotracheal tubes and respiratory secretions in an NICU of a referral teaching hospital in Greece between April and October 2002. None of the neonates progressed to clinical infection, and none of them received specific treatment. This study suggested that *C. meningosepticum* colonization in neonates does not necessarily lead to infection and that such colonization outbreaks may be controlled with emphasis on the standard precautions [22]. 

 Hsu et al. [34] studied a total of 118 patients with *C. meningosepticum* bacteremia at a medical center in Taiwan from 1999 to 2006. Among 99 preserved isolates, 84 % presented with fever, 86% had nosocomial infections, and 60% had acquired the infection in intensive care units (ICUs). Seventy eight percent of patients had primary bacteremia, followed by pneumonia (9%) and catheter-related bacteremia (6%). Forty-five patients (38%) had polymicrobial bacteremia. Only 6 patients were under the age of 18 and none of them was premature, a much lower percentage than in previous reports [34]. 

 We [16] reported three clusters of *C. meningosepticum* infections in our hospital in July 2006 and January 2007 involving 8 newborns and 5 older children. Seven of the newborns were premature. The index patient was from the neonatal intensive care unit, and the older patients were from other pediatric wards. Three of them had meningitis, two had primary bacteremia, five had sepsis, one had postoperative cellulitis and fasciitis, and two had respiratory distress and pneumonia. The organism was isolated from the blood of all, cerebrospinal fluid (CSF) of 4 of the patients. Nine patients improved on antimicrobial treatment, and 4 premature infants died after the infection. One of the neonates who died had meningitis, one had sepsis, and the other two had respiratory distress and pneumonia [16].

## 6. Infection Control Interventions

 Outbreaks of Gram-negative bacterial infections are usually due to transient carriage of the organisms on the hands of healthcare workers [35]. Susceptible patients may become colonized after acquiring the organism from the healthcare worker, and infection may or may not develop.

 Gram-negative bacteria can have an inanimate reservoir such as hospital sinks [35, 36]. It is generally considered that this is not an important factor in endemic hospital-acquired infections. In those susceptible to infection, however, small numbers of potentially pathogenic organisms present on healthcare workers' hands, after washing them in contaminated tap water, may cause infection [12, 35].

 To detect the source of an outbreak of *C. meningosepticum*, it is important to obtain cultures from food and infant formulas, wet areas, dry surfaces, equipments, and the hands of healthcare workers who contact with the patients. Obtaining cultures on a periodic basis is necessary [18].

 Measures that have been used to eradicate *C. meningosepticum* outbreaks in pediatric wards include changing the prescribing policy for empiric antibiotics, restriction of further admissions, and thorough disinfection of the unit [9, 11, 15]. Other studies have shown successful control with milder measures, including alcoholic hand rub after the washing of hands, toileting of babies with sterile instead of tap water, and repairing, cleaning, super chlorination, isolation of the water tanks from all the hospital feeder tanks and changing the sink taps [11, 37, 38]. Discarding opened creams, ointments, sterile water, and hand-washing solutions have also been among other interventions of infection control. Strict supervision of the hygiene during the preparation process of intravenous lipid solutions as well as other intravenous solutions and the infant formulas is also important as these have also been reported as important sources of epidemics [13, 16, 18]. In our experience, we determined that electrical buttons, computer keyboards, phones, doorknobs, and ambu bags may also be sources of epidemics [16]. Particularly in intensive care units, the frequent dysinfection of these items as well as hand hygiene can prevent epidemics. Restriction of staff exchange between wards; isolation of the patients with *C. meningosepticum* positive cultures; scrubbing units and wards thoroughly using two disinfectants (hypochlorite solution and isopropanol spray) with special emphasis on objects containing or in contact with water have shown successful control [39]. In-service training should be implemented to reemphasize handwashing and contact precautions to staff. 

 In conclusion, surveillance for the reservoir and maintenance of rigorous infection control measures are essential to control *C. meningosepticum* outbreaks in the hospital setting.

## 7. Resistance Patterns and Mechanisms

*C. meningosepticum* has unusual resistance patterns and mechanisms. *Chryseobacteria* are resistant to multiple antibiotics, especially to *β*-lactams. Many possess two different types of *β*-lactamases, namely class A extended-spectrum *β*-lactamases and class B metallo-*β*-lactamases (MBLs); the latter confer resistance to carbapenems, which are widely used to treat infections caused by multidrug-resistant Gram-negative bacteria. Two types of MBL, BlaB and GOB, have been identified in isolates of *C. meningosepticum*. Although they have similar molecular weights, these two enzyme types show only very low molecular similarity. Sequencing and analysis of genes encoding BlaB and GOB has revealed heterogeneity, with up to 12 blaGOB and 14 blaB alleles identified [40]. *C. meningosepticum *is intrinsically resistant to most *β*-lactams, including carbapenems, due to production of chromosomal metallo-*β*-lactamases (MBLs) [41]. Bellais et al. reported that all *C. meningosepticum *isolates harbored two types of MBLs simultaneously: BlaB belonging to subclass B1 and GOB belonging to subclass B3 [42]. However, a recent survey in China reported that only 55 of 170 *C. meningoseptica *isolates harbored both types of MBLs, and 38 isolates harbored only one type of MBL [43]. Furthermore, no MBL genes were detected in the remaining 77 isolates, even though many of these isolates were resistant to imipenem. PCR experiments detected both genes encoding BlaB and GOB MBLs in all *C. meningosepticum *isolates. DNA sequence analysis revealed that *C. meningosepticum *isolates possessed 7 types of *bla*B gene, including 5 novel variants (*bla*B-9 to *bla*B-13), and 11 types of *bla*GOB gene, including 10 novel variants (*bla*GOB-8 to *bla*GOB-17). The most common combination of MBLs was BlaB-12 plus GOB-17 [43].

 These organisms are generally resistant to the conventional chemotherapeutic agents used in the treatment of neonatal meningitis, such as ampicillin, gentamicin, kanamycin, and chloramphenicol [32, 44]. Different resistance patterns have been reported in different outbreaks worldwide.  Table 1 shows the susceptibilities of the strains of *C. meningosepticum* isolated during the outbreaks reported in the literature.

## 8. Treatment

 The appropriate choice of antimicrobial agents effective for the treatment of chryseobacterial infections is quite difficult to make. *Chryseobacterium spp.* are resistant to most antibiotics and the use of inactive drugs as empirical therapy may contribute to the poor outcome in many infections. In addition, MIC breakpoints have not been established by the National Committee for Clinical Laboratory Standards (NCCLS) for *Chryseobacteria* [23]. Antimicrobial susceptibility data on *Chryseobacterium spp.* remain very limited, since this pathogen has been rarely isolated from clinical specimens. In addition, results of susceptibility testing vary when different methods are used. Results from disk diffusion methods may not be reliable, so broth reference quality microdilution tests should be performed when possible. The *E*-test has also been suggested as a possible alternative for testing certain antibiotics [5].

*Chryseobacterium* organisms produce *β*-lactamases and are resistant to most *β*-lactam drugs, including the carbapenems and aztreonam. Cefepime has poor activity against *C. meningosepticum*. These organisms have an unusual pattern of antibiotic sensitivity, being generally resistant to the conventional chemotherapeutic agents used in the treatment of neonatal meningitis, such as ampicillin, gentamicin, kanamycin, and chloramphenicol [30, 44].

 Hoque et al. [9] reported a strain of multiresistant *C. meningosepticum *isolated from eight neonates on a neonatal intensive care unit from September 1994 to May 1996. The strain was resistant to ampicillin, ceftazidime, imipenem, gentamicin, ciprofloxacin, and trimethoprim sulfamethoxazole, susceptible to piperacillin and amikacin, and had variable susceptibility to rifampicin and vancomycin.

 Güngör et al. [11] reported that although all of their isolates in the outbreak were susceptible to ciprofloxacin *in vitro*, three patients did not respond to ciprofloxacin therapy given for 6 or 7 days. They switched the therapy to vancomycin and rifampin, and all three patients survived with one having a complication (hydrocephalus). 

 According to the results of the SENTRY Antimicrobial Surveillance Program, *Chryseobacterium spp.* are known to exhibit resistance to aminoglycosides, tetracyclines, chloramphenicol, erythromycin, clindamycin, and teicoplanin [6, 45, 46]. However, some fluoroquinolones have shown favorable results [47, 48]. Sparfloxacin, clinafloxacin, and levofloxacin are somewhat more active than ciprofloxacin [49]. Minocycline has also shown good in vitro activity, while susceptibility to doxycycline and trimethoprim sulfamethoxazole appears variable. Rifampin is usually active in vitro and has been used as part of a combination therapy to clear persistent infection [50]. Vancomycin alone or in combination with other agents, including rifampin, has been successful in the treatment of meningitis in infants [51]. However, the usefulness of vancomycin against *Chryseobacterium spp.* infections has more recently been questioned [45, 47]. It was reported that vancomycin was inactive in vitro (MIC of 16 to more than 64 *μ*g/mL).

 In our experience [18], although all of our isolates remained susceptible to vancomycin, rifampicin, and linezolid during the outbreak, resistance to imipenem and amikacin was increased in the second and third clusters. This may be due to an inducible resistance against these antibiotics. The possibility of this type of resistance should be considered when choosing an antibiotic regimen to treat *C. meningosepticum* infection. Four of our 13 cases (1 in the second cluster and 3 in the third cluster) died despite antibacterial treatment, which had appeared to be effective in sensitivity testing. This may be due to the neonates' prematurity and severe disease and also may represent a difference of in vitro activity. 

 Thus, there is no optimal regimen for the treatment of *Chryseobacterium spp.* İnfections, and antimicrobial therapy should be based on MIC results from properly performed susceptibility tests.

## 9. Prevention

 Clinical studies demonstrate the importance of rapid instigation of epidemiological investigation, whilst also ensuring that fundamental infection control procedures are in place during an outbreak and to prevent an outbreak. It is worthwhile for the infection control team to ensure that the hospital water tanks are inspected and chlorinated yearly and any necessary repair work carried out. We could not find any study for vaccine development in the literature.

## Figures and Tables

**Table 1:** The susceptibilities of the strains of *C. meningosepticum. *

	Resistance/susceptibility			
References	Aminoglycosides	Beta-lactams	Glycopeptides	Quinolones
Hazuka et al. 1977 [15]	NA	R	R	NA
Thong et al. 1981 [14]	R	R	NA	NA
Di Pentima et al. 1998 [33]	IR	R	R	S
Chiu et al. 2000 [7]	R	R	NA	IR
Hoque et al. 2001 [9]	R	R	R/S	R
Güngör et al. 2003 [11]	R	R	S	S
Ceyhan et al. 2008 [16]	R	R	S	S
Maraki et al. 2009 [22]	IR	R	NA	NA
Hsu et al. 2010 [34]	R	R	NA	S

R: resistant, IR: intermediately resistant, S: susceptible, NA: data not available.
